# Supplementary figures and images for: Molecular identification of Hymenopteran insects collected by using Malaise traps from Hazarganji Chiltan National Park Quetta, Pakistan
Source: PLoS One. 2024 Apr 10;19(4):e0300903. doi: 10.1371/journal.pone.0300903 (PMC11006193; doi:10.1371/journal.pone.0300903)

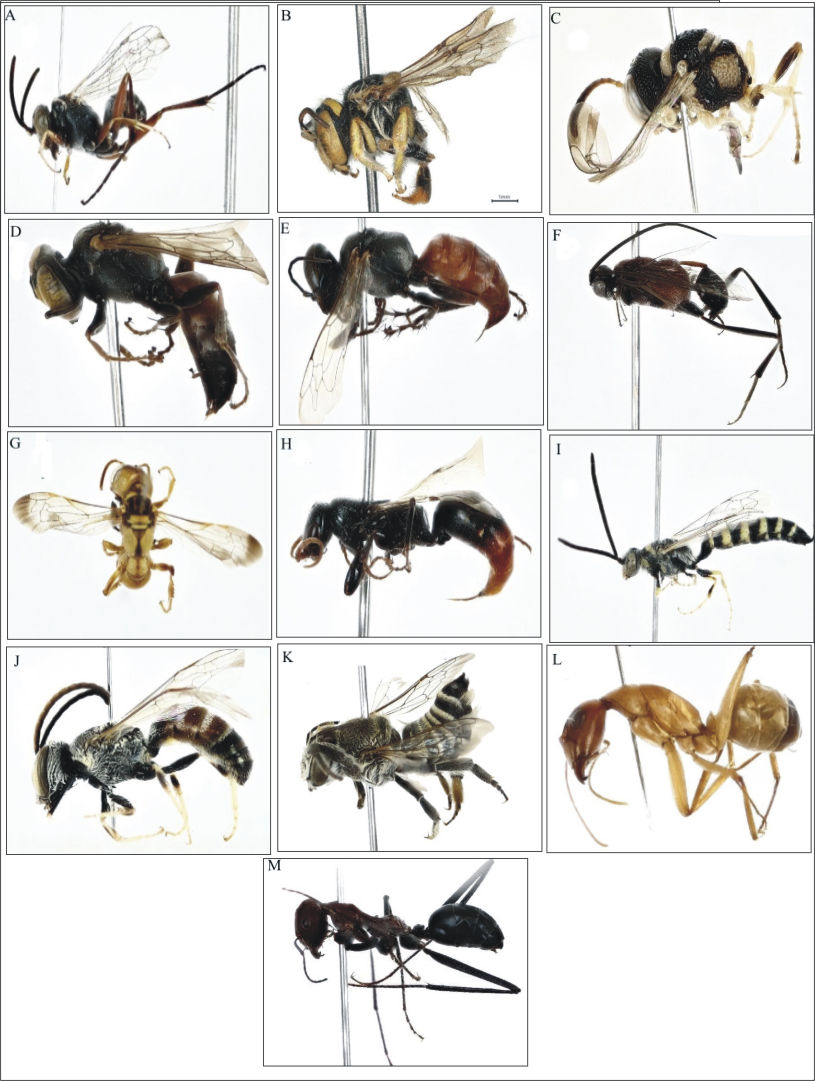

Supplement: S1 Fig — Figure presents a taxonomically arranged list of morphologically identified Hymenopterans insects: (A) Anthidium punctatum (B) Bethylidae sp. (C) Camponotus compressus (D) Ceropales seyrigi (D) Evaniidae sp. (F) Formicidae sp. (G) Lasioglossum sp. (H) Megachile leachella (I) Hymenoptera sp. (J) Sphecidae sp. (K) Tachysphex incertus (L) Tachysphex sp. The datasets presented in the study have been deposited to the NCBI GenBank repository (ncbi.nlm.nih.gov), and the obtained accession numbers are OR923389 (Cerceris rybyensis), OR924175 (Megachile leachella), OR923376 (Anthidium punctatum), OR924115 (Camponotus compressus), OR924210 (Tachysphex incertus), OR924219 (Tachytes freygessneri), OR939445 (Tachysphex sp.), OR924342 (Lasioglossum sp.), OR924339 (Bethylidae sp.), OR924340 (Formicidae sp.), OR924341 (Evaniidae sp.), OR939444 (Sphecidae sp.), and OR939995 (Hymenoptera sp.). (TIF) [file pone.0300903.s001.tif]
