# Supplementary material for: Molecular identification of Hymenopteran insects collected by using Malaise traps from Hazarganji Chiltan National Park Quetta, Pakistan
Source: PLoS One. 2024 Apr 10;19(4):e0300903. doi: 10.1371/journal.pone.0300903 (PMC11006193; doi:10.1371/journal.pone.0300903)
Supplement: Attachment — Submitted filename: ploseone review.docx [file pone.0300903.s002.docx]

**Title**

**ok**

**Introduction**

1. Summarize first paragraph, its too long to just describe insect and insect orders.
2. Line 92-101 .Please a lot of other studies have been done in Pakistan on Order Hymenoptera. Please update this paragraph.
3. This whole part carry lot of information please be precise according to the title.

**Methods and Materials**

1. Line 116 please add relevant citation.
2. Line 129-132 please add citation to the methodology followed.
3. Line 142-147. This information is not sufficient. How different groups of Order Hymenoptera can be identified by a few stander keys. All groups how can be identified by few characters (physical features such as color pattern, wing venation, body shape, antennae and head).Please add typical characters and exact keys for the identification of all included groups of insects.
4. Line 160-169 need proper citation.

**Results**

1. Morphological identification

As your title indicates this study also included morphological identification too then please add main identification characters of all identified species along with the colored photographs of main identification characters. Just to give the names of species is not sufficient and seems suspicious.

2. Line 211-212 (This data indicates that the distribution of Hymenopteran insects follows a Gaussian distribution) please discuss how and why in discussion part too..

**Discussion**

This part needs a lot of improvement.

1. Please discus all your results one by one firstly with the morphological identification results and then come to molecular identification results supported with most relevant results of other studies. Authors have just given general statements (as in the first paragraph) in the support of their results which need to be rectified.
2. Only in this part authors have again repeated results rather than to discuss them.
3. Most of this part has been discussed with irrelevant discussions.
4. Discussion on the phylogenetic tree (Figure 4) is totally missing.
5. Quality of all figures is very poor. There is a need of high quality figures here.

**General Comments**

Although the results of the study are admirable and would provide basis for further studies. The manuscript need a lot of improvements prior to publication.
