# Supplementary material for: Molecular identification of Hymenopteran insects collected by using Malaise traps from Hazarganji Chiltan National Park Quetta, Pakistan
Source: PLoS One. 2024 Apr 10;19(4):e0300903. doi: 10.1371/journal.pone.0300903 (PMC11006193; doi:10.1371/journal.pone.0300903)
Supplement: Attachment — Submitted filename: Response to Reviewers.docx [file pone.0300903.s003.docx]

To

Editor **“***PLOS ONE*”

**Dear Sir \ Madam,**

We would like to re-submit our revised manuscript, entitled “**Molecular identification of Hymenopteran insects collected by using Malaise traps from Hazarganji Chiltan National Park Quetta, Pakistan**”, and the suggestions as well as recommendations were added in this revised manuscript. We greatly appreciate your time for handling our submission and assessing the review comments. The issues raised by the reviewers have been addressed in this revised MS.

Profound regards!

**Muhammad Kashif Obaid** (PhD)

**WhatsApp**: +86-18500565541

**Email**: [kashifobaidkanz@gmail.com](mailto:kashifobaidkanz@gmail.com)

Asmatullah Kakar (PhD)

**Email**: [asmardanzai@yahoo.com](mailto:asmardanzai@yahoo.com)

**Editor’s # comments**

Please include the following items when submitting your revised manuscript:

**Comment-1.** A rebuttal letter that responds to each point raised by the academic editor and	reviewer(s). You should upload this letter as a separate file labeled 'Response to Reviewers'.

**Author’s response**: We have prepared the rebuttal letter and as per suggestion is labeled.

**Comment-2.** A marked-up copy of your manuscript that highlights changes made to the original version. You should upload this as a separate file labeled 'Revised Manuscript with Track Changes'.

**Author’s response:** We have made all changes as suggested by the reviewers, and the changes in the revised manuscript have been highlighted in the ‘track changes’.

**Comment-3.** An unmarked version of your revised paper without tracked changes. You should upload this as a separate file labeled 'Manuscript'.

**Author’s response:** We have attached two files as suggested: one showing the tracked changes and one with the changes applied.

**Comment-4.** If you would like to make changes to your financial disclosure, please include your updated statement in your cover letter. Guidelines for resubmitting your figure files are	available below the reviewer comments at the end of this letter.

**Author’s response:** We do not want to modify our financial statement because this research was conducted without any financial support.

**Journal Requirements**:

When submitting your revision, we need you to address these additional requirements.

**Comment-1.** Please ensure that your manuscript meets PLOS ONE's style requirements, including those for file naming: Did you know that depositing data in a repository is associated with up	to a 25% citation advantage (<https://doi.org/10.1371/journal.pone.0230416>)? If you’ve not	already done so, consider depositing your raw data in a repository to ensure your work is	read.

**Author’s response:** We have successfully submitted our sequencing data to both NCBI (GenBank) and BOLD-database, and we have included the details pertaining to this data in the Supporting Information section of our manuscript.

**Comment-2.** We suggest you thoroughly copyedit your manuscript for language usage, spelling, and grammar.

**Author’s response:** We are grateful for the observation of this comment. An extensive revision of this MS through Professor Dr. Muhammad Ashfaq Ahmed (University of Balochistan, Quetta) has been conducted.

**Comment-3.** Please take this opportunity to be sure you have met all of our guidelines for new species. For proper registration of a new zoological taxon, we require two specific statements	to be included in your manuscript.

**Author’s response:** Thank you to the reviewers for drawing attention to this point. Nevertheless, it is important to mention that the report of *Anthidium punctatum* from Pakistan does not constitute a novel species: data of this specie was already available in GenBank and on BOLD databases. For this reason, there is no need to include a subsection on "Nomenclatural Acts" in the Methods section. .

**Comment-4.** We note that you have stated that you will provide repository information for your data at acceptance.

**Author’s response:** Thank you for your comment. We have successfully obtained accession numbers for all sequences from GenBank and BOLD IDs. However, to ensure data preservation, we have opted to release the accession numbers to GenBank upon acceptance of this manuscript (before final proof read). Additionally, we have updated the statement regarding this in the revised MS.

**Comment-5.** Please amend the manuscript submission data (via Edit Submission) to include author "Zafarullah".

**Author’s response:** Amended the author name as per suggestion.

**Comment-6.** We note that Figure 1 in your submission contain map images which may be copyrighted.

**Author’s response:** We acknowledge your feedback. To ensure scientific credibility, we have indicated in the revised MS that the figure (1) was generated using ArcGIS software and was not copied from any external sources. Further, the link to the original software is also provided in the revised MS. This same software had been previously used to develop the figure and the corresponding research has been published in PLOS ONE (https://doi.org/10.1371/journal.pone.0268801).

**Comment-7.** We notice that your supplementary figure 2 are uploaded with the file type 'Figure'. Please amend the file type to 'Supporting Information'. Please ensure that each Supporting Information file has a legend listed in the manuscript after the references list.

**Author’s response:** Correction are made in supplementary figure 2.

**Reviewer’s Comments**

**Comment-1.** Title

**Author’s response:** We have revised the title according to the reviewer's recommendations.

**Comment-2.** Introduction

- Summarize first paragraph, its too long to just describe insect and insect orders.
- Line 92-101 .Please a lot of other studies have been done in Pakistan on Order Hymenoptera. Please update this paragraph.
- This whole part carry lot of information please be precise according to the title.

**Author’s response:** All the above suggestions in introduction section has been made and inserted in the revised manuscript.

**Comment-3.** Methods and Materials

- Line 116 please add relevant citation.
- Line 129-132 please add citation to the methodology followed.
- Line 142-147. This information is not sufficient. How different groups of Order Hymenoptera can be identified by a few stander keys. All groups how can be identified by few characters (physical features such as color pattern, wing venation, body shape, antennae and head).Please add typical characters and exact keys for the identification of all included groups of insects.
- Line 160-169 need proper citation.

**Author’s response:** Revisions have been made to the manuscript based on the provided suggestions and recommendations.

**Comment-4.** Results

- Morphological identification
- As your title indicates this study also included morphological identification too then please add main identification characters of all identified species along with the colored photographs of main identification characters. Just to give the names of species is not sufficient and seems suspicious.
- Line 211-212 (This data indicates that the distribution of Hymenopteran insects follows a Gaussian distribution) please discuss how and why in discussion part too.

**Author’s response:** Thank you for commenting on this part. As the primary focus of this study was to molecularly identify various Hymenopteran insects, hence we did not detail the morphology of these insects, instead we compared them to various standard keys cited in the text. To promote clarity, the title of this manuscript has been modified accordingly. Additionally, the discussion section includes a discussion of Gaussian distribution.

**Comment-5.** Discussion

This part needs a lot of improvement.

- Please discus all your results one by one firstly with the morphological identification results and then come to molecular identification results supported with most relevant results of other studies. Authors have just given general statements (as in the first paragraph) in the support of their results which need to be rectified.
- Only in this part authors have again repeated results rather than to discuss them.
- Most of this part has been discussed with irrelevant discussions.
- Discussion on the phylogenetic tree (Figure 4) is totally missing.
- Quality of all figures is very poor. There is a need of high quality figures here.

**Author’s response:** In response to comment 4, the results have been removed from the discussion section and the discussion section has been thoroughly revised. A phylogenetic tree has been included in the discussion section. High quality figures have been included in this revised version of the manuscript.

**Comment-6.** General Comments

Although the results of the study are admirable and would provide basis for further studies. The manuscript need a lot of improvements prior to publication.

**Author’s response:** Thank you reviewers for the valuable comments. We have worked in the revised MS and updated accordingly.
